# Resting-State fMRI in MS: General Concepts and Brief Overview of Its Application

**DOI:** 10.1155/2015/212693

**Published:** 2015-08-27

**Authors:** Emilia Sbardella, Nikolaos Petsas, Francesca Tona, Patrizia Pantano

**Affiliations:** ^1^Department of Neurology and Psychiatry, Sapienza University of Rome, Viale dell'Università 30, 00185 Rome, Italy; ^2^IRCSS Neuromed, Via Atinense 18, 86077 Pozzilli, Italy

## Abstract

Brain functional connectivity (FC) is defined as the coherence in the activity between cerebral areas under a task or in the resting-state (RS). By applying functional magnetic resonance imaging (fMRI), RS FC shows several patterns which define RS brain networks (RSNs) involved in specific functions, because brain function is known to depend not only on the activity within individual regions, but also on the functional interaction of different areas across the whole brain. Region-of-interest analysis and independent component analysis are the two most commonly applied methods for RS investigation. Multiple sclerosis (MS) is characterized by multiple lesions mainly affecting the white matter, determining both structural and functional disconnection between various areas of the central nervous system. The study of RS FC in MS is mainly aimed at understanding alterations in the intrinsic functional architecture of the brain and their role in disease progression and clinical impairment. In this paper, we will examine the results obtained by the application of RS fMRI in different multiple sclerosis (MS) phenotypes and the correlations of FC changes with clinical features in this pathology. The knowledge of RS FC changes may represent a substantial step forward in the MS research field, both for clinical and therapeutic purposes.

## 1. Introduction

Multiple Sclerosis (MS) is an inflammatory and degenerative disease of the central nervous system (CNS). It is characterized by multiple lesions mainly affecting the white matter, with consequent structural and functional disconnection between various areas of the CNS, resulting in a wide range of signs and symptoms.

Functional magnetic resonance imaging (fMRI) during the performance of various tasks has provided a large amount of data showing functional alterations in MS patients, generally interpreted as adaptive plastic changes aimed at limiting the clinical impact of the disease [1, 2]. More recently, fMRI studies during the resting-state (RS) allowed exploring the functional connectivity (FC) of the brain. This aspect is of particular interest in MS, which is considered among the disconnection syndromes [3, 4]. The study of RS FC in MS is mainly aimed at understanding alterations in the intrinsic functional architecture of the brain and their role in disease progression and clinical impairment. RS fMRI can be used to identify anatomically separate, though functionally connected, brain regions configuring specific RS networks [5]. Unlike fMRI during task execution, RS fMRI is not influenced by task performance, which may differ from that of healthy subjects, especially in patients with clinical disability.

In this brief review we are going to explain the physiological aspects underlying brain RS FC and to describe the methodological approaches to analyze it. We will then focus on the applications of RS fMRI in various phenotypes of MS, also considering the correlations between clinical impairment and both within- and between-network FC alterations in MS. Functional changes do not necessarily represent adaptive neuroplasticity aimed at maintaining a normal function despite widespread CNS pathological involvement; in some instances they could represent an inefficient or even worsening attempt to compensate for tissue damage, that is, maladaptive plasticity. Correlations between changes in FC and the level of clinical impairment could help to distinguish between beneficial and nonbeneficial neuroplastic changes.

Lastly, we briefly expose some of the most promising directions for further investigation of RS FC in MS.

## 2. Resting-State fMRI: Physiological Bases

Brain activity has usually been considered as a response to external and internal stimuli, though organized activity has also been demonstrated at rest. Resting-state functional magnetic resonance imaging (RS fMRI) is used to analyze functional coherence in the activity of different brain areas, that is, functional connectivity, at rest (RS FC). This technique detects spontaneous low-frequency fluctuations (approximately in the domain of 0.01–0.1 Hz) of the blood-oxygen-level-dependent (BOLD) signal [6] that are temporally coherent across anatomically separated networks (RSNs) [7] and that represent well-organized brain activity [8]. The BOLD signal, on which fMRI is based, is due to changes in the concentration of deoxygenated hemoglobin, an endogenous paramagnetic contrast agent [9], which results in a decrease in the local magnetic field that can be detected on T2-weighted Echo-Planar imaging [6]. When a brain area is activated, cerebral blood flow and velocity increase to a greater extent than O_2_ extraction [10, 11], thereby raising the blood oxygenation level, which in turn increases the MRI signal. The BOLD signal reflects specific biological and functional events and is believed to be due to the increased neural activity caused by a combination of biological mechanisms, including effects from neurotransmitters, ions, and other metabolites [12–14]. Nevertheless, whether BOLD signal fluctuations represent changes in brain physiology that are independent of neuronal function [15–17] or reflect neuronal baseline activity [18, 19] is not yet clear. Some studies suggest that RS fluctuations are an intrinsic property of the brain since they persist across conditions such as sleep [20], anesthesia [21], and task execution [22]. On the other hand, the neuronal origin of BOLD activity is supported by studies based on a combination of fMRI and positron emission tomography (PET), which highlighted the involvement of the grey matter (GM) alone in significant voxels [23], by studies based on a combination of fMRI and electroencephalograms, which revealed a correlation between BOLD signal and cortical electrical activity [24, 25], and by studies that highlighted RSNs changes induced by neurological disease [26].

## 3. Resting-State fMRI: Methodological Approaches

**Figure 1:**
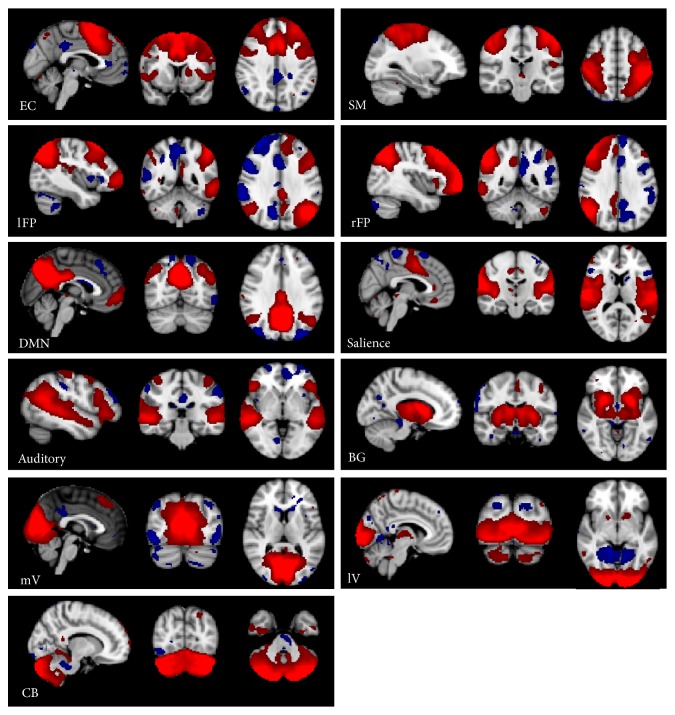
Eleven resting-state networks identified by using independent component analysis (use of MELODIC tool by FMRIB Software Library toolbox, on a cohort of 20 healthy subjects, elaboration on our data) one sample* t*-test, (*P* < 0.05, family-wise corrected). Red shows positively correlated voxels and blue shows negatively correlated voxels. fMRI results are overlaid on the MNI152, 1 mm, standard brain. Images are shown according to the radiological convention. EC: executive control; SM: sensory-motor; lFP-rFP: left and right frontoparietal; DMN: default mode network; lV: lateral visual; mV: medial visual; CB: cerebellum; BG: basal ganglia.

To provide the best possible setting for RS studies, subjects are usually instructed to stay awake, calm, and still in the scanner, to fix a specific point or close their eyes, and to try not to think about anything. The use of a high magnetic field is usually better, since it would allow to more easily detect signal changes, which are proportional to the main magnetic field, and to separate noise frequencies from proper RSNs more effectively due to a short relaxation time [11]. The aim of the fMRI application is to detect different RSNs and to investigate their involvement in specific functions. The two most commonly applied methods for RS investigation are the region-of-interest (ROI) analysis and the whole brain investigation, the latter consisting mainly of the independent component analysis (ICA) [27]. The ROI analysis correlates the time course of a predefined ROI with other brain voxels [7, 8], according to the detection of coherent BOLD fluctuations. However, this approach is limited by the relative arbitrariness of the ROI selection. Conversely, ICA is a data-driven, whole-brain approach [27–29], designed to separate a multivariant signal in its sub-components, thus providing a single signal from a complex of signals. ICA is used without any* a priori* hypothesis and assuming the statistical independence of the sources and the BOLD signal is decomposed into spatially and temporally distinct maps with their own time courses. Each map may be interpreted as a network of brain regions that share similar BOLD fluctuations over time. One issue that needs to be considered when detecting RSNs, using either regional or whole brain analysis, is the presence of possible artifacts related to movement and to physiological noise, that is, cardiac and respiratory cycles [5]. Nevertheless, a frequency difference has been demonstrated between RSNs and noise, with the former being characterized by fluctuations of 0.01–0.1 Hz and the latter by fluctuations of 0.3–1 Hz [5]. Given the importance of removing confounding signals to improve the quality of the data [17, 30, 31], noise signals are now commonly monitored by means of specific software that retrospectively corrects the fMRI data [32]. Similarly, other sources of regionally specific noise, such as white matter (WM) and cerebrospinal fluid (CSF) signals, should be considered and removed during the analysis [33], as the BOLD signal in these regions is more susceptible to artifact than in cortical GM [34]. Despite all the technical issues that are involved in the collection of RS BOLD data, no consensus has yet been reached on the need for a precise experimental setting [20, 35]. Nevertheless, the detection of many neuroanatomical systems whose spontaneous activity is consistent has led to the identification of specific functional RSNs [5, 8, 36]. The best known of these systems are the default mode, sensory-motor, dorsal attention, visual, executive function, auditory, lateralized frontoparietal, salience, cerebellar, and basal ganglia networks [5, 37] (see Figure 1). Recently, changes in FC metrics over time have been also demonstrated, thus giving rise to the characterization of dynamic FC [38]. Emerging literature, by using new techniques of analyses, that is, sliding-window analysis, time-frequency coherence analysis, and flexible least squares based time-varying parameter regression strategy [38, 39], suggests that dynamic FC metrics may provide existence of changes in macroscopic neural activity patterns likely related to behavioral conditions [40]. However, limitations related to analysis and interpretation remain and it is yet unclear whether dynamic FC consists of the recurrence of multiple discrete patterns or it is a simple pattern variation along time [38].

Brain function is widely known to depend not only on the activity of individual regions, but also on the functional interaction of different areas across the whole brain through the so-called connectomes [41–43]. Connectomes are axonal projections that allow functional communication between anatomically separate brain regions. Recent processing techniques enable the investigation of large-scale functional connections, thereby allowing the creation of a matrix graph of brain connectivity. Large-scale network connectivity is usually represented as a graph consisting of brain regions (nodes) that are interconnected (edges). Very briefly, after an initial definition of nodes, a matrix of functional connections between nodes is computed, though only connections higher than the setup threshold are classified as edges. Functional connectivity is provided as a statistical correlation coefficient of BOLD signal coherence between different networks [41]. The structure of a network may be designed according to the characteristics of certain graph values, such as the clustering-coefficient, the path length, the centrality, the degree, and the modularity of a node, thereby highlighting a specific organization pattern [44]. It has been demonstrated that global brain network connectivity presents a small-world organization that is far from random, characterized by a high level of local connections between nodes and a very short path length which configure the so-called “hub” and a low presence of long connections between hubs; this network organization elevates efficiency and reduces substantially redundancy [44]. The so called “rich-club” organization has been also demonstrated, consisting in the presence of more densely connected high-order hubs [45]. The rich-club phenomenon provides important information on the higher-order structure of a network, particularly on hierarchy and specialization [45].

Neurological pathologies may change node interactions, thereby disrupting the integration of systems and impairing their functioning.

## 4. Resting-State fMRI: Application in Multiple Sclerosis

Advances in the comprehension of FC and the role of its alterations in the pathophysiology of human brain are given by the study of disease like MS. In fact, MS is characterized by a particularly widespread and severe damage mainly affecting the white matter that can cause FC alterations secondary to structural disconnection between RSN nodes.

RSNs abnormalities have been found in almost all multiple sclerosis (MS) phenotypes [46–57].

FC is greater in specific brain areas of many RSNs in patients with clinically isolated syndrome (CIS) than in either healthy subjects (HS) or relapsing-remitting MS (RR-MS) patients, even though GM volume and WM integrity are preserved [46]. These results suggest that the coherence of cerebral activity increases in the earliest stage of the disease, probably as a compensatory phenomenon, and is subsequently lost in the late phase of the disease as a result of structural damage progression. However, an agreement on the actual meaning of fMRI changes in early MS has not yet been reached: even if the compensatory hypothesis is still prevailing, a single study reported lower global values of temporal coherence in CIS patients [47].

Results by RS fMRI were only partially concordant when RR MS subjects were studied [46, 48–51], probably because of the wide spectrum of clinical characteristics that are peculiar to this phenotype as well as of the different methodological approaches. Widespread FC abnormalities were found in RR-MS subjects: some studies pointed to a significant increase in global connectivity levels [46, 50, 52, 53] and others reported FC decrease [49, 51]. The FC reduction is in line with results from PET and MRI perfusion studies, which have shown diffuse brain hypometabolism [58, 59] and hypoperfusion [60] in this condition, probably due to the progressive accumulation of structural damage. The FC increase instead is a more complex event; although it is generally considered as an adaptive attempt to compensate for tissue damage, any alternative hypothesis that FC increase may represent maladaptive plasticity or an epiphenomenon of the pathological process cannot be completely ruled out [46, 48, 51, 54, 61, 62]. Lastly, some studies found that specific networks, that is, the thalamic RSN and DMN, may show both significantly weaker connections with some brain regions and stronger connections with others, thus suggesting that there is a redistribution of connectivity, besides a general trend of globally increased or decreased FC in MS [51, 55].

Only few studies focused on progressive MS phenotypes [56, 57]. In a recent work that explored FC alteration in RR and secondary progressive (SP) MS, authors found an increased FC in both groups of patients; however, specific changes in either direction were observed also between RR and SP MS groups. Interestingly, these FC changes seem to parallel patients' clinical state and capability of compensating for the severity of clinical/cognitive disabilities, supporting the compensatory role of functional reorganization [56].

In a study including patients with primary progressive (PP) and SP MS patients, compared to HS, FC was found to be decreased in some areas of the DMN in both groups of patients; FC in the anterior components of the DMN was correlated with cognitive impairment. When patients with SP and PP MS were compared, a higher FC in the anterior cingulate cortex was found in SP [57].

Taken together these results show that there is not a straightforward relationship between RSNs changes and clinical phenotype, suggesting a decisive role of specific clinical and genetic characteristics of single subjects in determining the functional response to the disease.

## 5. fMRI Functional Connectivity Changes and Their Correlation with Clinical Disability

### 5.1. Within-Network Connectivity

Correlations of within-network FC changes with clinical MS parameters have been widely reported in MS [46, 49, 50, 52, 57–59, 63]. Although the ability of RS fMRI to detect brain functional reorganization in MS has been proved, the role of FC alterations in the pathogenesis of MS, as well as the potential relationship between resting-state network reorganization and clinical disability, remains not completely understood.

A negative correlation between FC strength and clinical impairment has been repeatedly reported [46, 49, 50, 58, 59, 63]; few studies reported a positive correlation between FC strength and clinical impairment [52, 57]. Discordant results between studies may be due not only to differences in patient populations and data analysis, but also to the clinical function considered and the specific RSNs analyzed.

Regarding the correlations between the motor network and clinical disability, a recent work revealed an association between reduced intranetwork connectivity in the motor network and higher levels of disease severity in patients with RR MS, thus pointing to the possibility that resting-state changes may serve as a biomarker of disease progression [64]. On the other hand, increased connectivity in the left premotor area was found to be associated with greater clinical disability in RR MS though not in SP MS [52]. This finding suggests that even if disease progression is related to disrupted FC within the motor network, increased FC in specific motor areas may represent an attempt to compensate for the functional impairment, at least in RR MS.

Regarding correlations between FC alterations and cognitive performance, which results from the interaction of several complex brain functions involved in cognition, that is, working memory, attention, and executive function, the interpretation of results is more complex. Increased [65, 66], decreased [50, 57], and both increased and decreased [55] FC within sustained attention networks were found to be associated with cognitive performance in MS. FC decrease in the anterior components of the DMN was found to correlate with accumulation of cognitive deficits in patients with progressive MS [57]. Bonavita et al. [55] confirmed the anterior dysfunction of the DMN also in RR MS; moreover, they found that patients with RR MS also showed an increased FC in the posterior components of the DMN, which was more pronounced in cognitively preserved patients. A recent study on heterogeneous group of MS has shown that decreased cognitive performance is accompanied by reduced FC in all main RSNs and is also directly related to brain damage [67]. On the other hand, another study on RR MS, focused on the thalamic RSN, reported a decreased performance associated with increased FC, suggesting that neuroplastic changes are unable to fully compensate for cognitive dysfunction [51].

Taken together, these results demonstrate that RSN reorganization is closely associated with cognitive disability in MS. On the basis of this strong association, FC changes have been proposed as promising surrogate markers of disease burden [50] as well as useful tools to monitor rehabilitative strategies in MS. Indeed, cognitive rehabilitation has been shown to correlate with changes in the RS FC of brain regions subserving trained functions [68].

### 5.2. Large-Scale Network Connectivity

Studies of large-scale network connectivity have been applied in MS with the attempt to give a global view of distributed patterns of FC abnormalities also in relationship with structural damage and disability.

Abnormalities in the FC of large-scale networks have been demonstrated in MS patients, with the disconnection appearing to be proportional to the extent of the lesions and correlated with the severity of disability [69, 70]. The involvement of RSN disconnection in MS is widespread and includes motor, sensitive, visual, and cognitive network function abnormalities [41]. FC is usually decreased in the whole brain. For example, decreased FC in subcortical and cortical regions and contralateral connections has been shown to be related to lesion load and to be able to discriminate MS patients from controls with a sensitivity of 82% and specificity of 86% [63]. Furthermore, FC in attentional networks is stronger in cognitively preserved patients than in cognitively impaired patients and is correlated with lower structural damage [71]. Reduced functional integration between separate areas was also found in the early stages of MS [61]. These findings suggest that functional disconnection parallels both structural damage and clinical impairment.

By contrast, a higher degree of connectivity between RSNs associated with visual functions is correlated with higher disease burden in spite of reduced within-network connectivity in other areas [64]. This finding may be interpreted as a focused event within a framework of global reorganization of brain FC over the course of the disease. This hypothesis is supported by the finding of a widespread modularity redistribution in MS, with some RSNs displaying decreased connectivity, due in part also to lesion load and clinical impairment, and others displaying increased connectivity [72].

The large-scale connectivity analysis, when applied in patients in comparison to controls, may highlight the differences in the whole brain network functional organization between the two groups. Accordingly, large-scale FC has been proposed as a promising tool to discriminate MS subjects from HS, to understand the functional substrate of clinical disability, and to monitor the effects of therapies. However, further studies are needed to clarify the proper meaning of these changes and whether functional modifications limit the clinical impact of the disease or, conversely, are a biomarker of disease severity.

## 6. Discussion and Future Directions

fMRI technique allows to detect brain functional connectivity across the brain. Its application in neurological pathologies, that is, MS, may provide valuable information on the neuronal changes occurring after damage, thus helping to understand the pathophysiology of the disease and the possible therapeutic approaches. Widespread connectivity abnormalities are evident both within and between RSNs in MS patients, but, unfortunately, results are not always concordant and the meaning of fMRI changes in MS is not completely clear. Additionally, RS fMRI studies are limited by the interference of noise artifacts, such as respiratory or cardiac events, which may be partially responsible of these incongruences and may also explain, at least in part, discordant fMRI results in similar MS phenotypes across different studies [5, 30, 31]. Another issue that can affect the homogeneity of the results may be ascribed to the differences among patients, that is, in terms of disease duration, within the same cohort, or between cohorts with similar disease phenotype. This problem may also influence the correlations between FC results and behavioral measures, since they do not always show the same directions. Accordingly, the significance of fMRI alterations in neurological pathology, in terms of compensatory or maladaptive mechanisms, has yet to be clarified. Despite some discrepant results, an increased FC in RSNs has been repeatedly reported and interpreted as adaptive brain reorganization; this hypothesis is supported by the fact that increased RS connectivity in MS patients usually occurs in brain areas with extensive cortical connections [53]. However, this adaptive phenomenon may be a finite process that is present in the early stages of the disease but is lost in more advanced stages, when the structural damage and the clinical impairment are too severe to be compensated for. Indeed, the increase in functional coupling between some areas of the motor network that parallels the increasing disability appears to be limited to the RR stage of the disease and is lost in the more advanced stages [52]; similarly, FC in some regions of the DMN is higher in cognitively preserved than in cognitively affected RRMS patients [55, 71].

The RS dynamics characterization [38], the graph theoretical analysis to study brain network properties [45], and the integration of RS fMRI data with other techniques, that is, transcranial magnetic stimulation and PET, could provide in the next future new insight into the pathophysiology of MS for clinical and therapeutical purposes.
